# Cortical layers, rhythms and BOLD signals

**DOI:** 10.1016/j.neuroimage.2017.11.002

**Published:** 2017-11-03

**Authors:** René Scheeringa, Pascal Fries

**Affiliations:** a Donders Institute for Brain, Cognition and Behaviour, Radboud University Nijmegen, Kapittelweg 29, 6525 EN Nijmegen, The Netherlands; b Institut National De La Santé Et De La Recherche Médicale U1028, Centre National De La Recherche Scientifique UMR S5292, Centre De Recherche En Neurosciences De Lyon, Bron, France; c Ernst Strüngmann Institute (ESI) for Neuroscience in Cooperation with Max Planck Society, Deutschordenstraβe 46, 60528 Frankfurt, Germany

## Abstract

This review investigates how laminar fMRI can complement insights into brain function derived from the study of rhythmic neuronal synchronization. Neuronal synchronization in various frequency bands plays an important role in neuronal communication between brain areas, and it does so on the backbone of layer-specific interareal anatomical projections. Feedforward projections originate predominantly in supragranular cortical layers and terminate in layer 4, and this pattern is reflected in inter-laminar and interareal directed gamma-band influences. Thus, gamma-band synchronization likely subserves feedforward signaling. By contrast, anatomical feedback projections originate predominantly in infragranular layers and terminate outside layer 4, and this pattern is reflected in inter-laminar and interareal directed alpha- and/or beta-band influences. Thus, alpha-beta band synchronization likely subserves feedback signaling. Furthermore, these rhythms explain part of the BOLD signal, with independent contributions of alpha-beta and gamma. These findings suggest that laminar fMRI can provide us with a potentially useful method to test some of the predictions derived from the study of neuronal synchronization. We review central findings regarding the role of layer-specific neuronal synchronization for brain function, and regarding the link between neuronal synchronization and the BOLD signal. We discuss the role that laminar fMRI could play by comparing it to invasive and non-invasive electrophysiological recordings. Compared to direct electrophysiological recordings, this method provides a metric of neuronal activity that is slow and indirect, but that is uniquely non-invasive and layer-specific with potentially whole brain coverage.

## Introduction

Electrophysiological and hemodynamic measures are the two most prominent tools to study brain function non-invasively in humans. The two methodological approaches are thought to provide largely complementary information on how the brain functions. EEG and MEG recordings provide a direct measure of neuronal responses with millisecond resolution, but have a relatively poor spatial resolution and primarily reflect synchronized post-synaptic potentials in the apical dendrites of pyramidal neurons. FMRI on the other hand can inform us about where in the brain changes in neuronal activity occur with millimeter-level precision, while covering the entire brain or a large part of the brain. Hemodynamics-based techniques like fMRI however only provide an indirect measure of neuronal activity with a temporal resolution on the order of seconds.

Over approximately the past decade, invasive electrophysiological recordings in animals have demonstrated that neuronal rhythms in different characteristic frequency bands occur in patterns that are specific to cortical layers (Bollimunta et al., 2008, 2011; Buffalo et al., 2011; Maier et al., 2010, 2011; Spaak et al., 2012; van Kerkoerle et al., 2014; Xing et al., 2012) and that they entrain remote neuronal groups through specific anatomical projections (Bastos et al., 2015a, 2015b; Michalareas et al., 2016). Over the same time period, laminar fMRI has developed from a technical and methodological challenge (Goense and Logothetis, 2006; Koopmans et al., 2010, 2011; Polimeni et al., 2010) to a viable tool to study brain function (De Martino et al., 2015; Kok et al., 2016; Muckli et al., 2015). Furthermore, the strength of neuronal rhythms in various frequency bands has been found to be correlated with the strength of the BOLD signal, both in anesthetized cat and macaque (Logothetis et al., 2001; Niessing et al., 2005) and in awake human subjects (Scheeringa etal., 2011a).

The aim of this review is to illustrate how laminar fMRI can complement investigations of the role of neuronal rhythms in brain function. For this purpose, we have divided the review into three sections. In the first section, we will discuss several theories on the role of neuronal rhythms in brain function. In this section, we will focus on alpha-, beta- and gamma-band rhythms and their roles in neuronal communication, which are closely linked to cortical layers and their anatomical projections. In the second section, we will discuss how these separate frequency bands relate to the BOLD signal, based on both animal and human studies. Finally, in the third section, we will elaborate on how this link between neuronal rhythms and laminar fMRI might complement our understanding of the role of neuronal rhythms in neuronal communication.

## Layer-specific neuronal rhythms and brain function

Since the discovery of human alpha waves (≈8–12 Hz) by Hans Berger in 1929 (Berger, 1929), we know that brain activity is in part rhythmic in nature. Since this seminal work, neuronal rhythms with a wide spectrum of frequencies have been discovered and described (Keitel and Gross, 2016) and are now investigated with a wide variety of electrophysiological recording techniques in both animals and humans. In this section, we will focus on recent work that links synchronization in the alpha-, beta- and gamma-frequency bands to laminar anatomical projection patterns, and that suggests distinct roles for these rhythms in the communication between cortical areas.

As mentioned above, the alpha-band rhythm was the first brain rhythm observed in human EEG recordings. The alpha rhythm is strong when brain regions are inactive. It has therefore been proposed that the alpha rhythm actively inhibits task-irrelevant brain regions (Jensen and Mazaheri, 2010). A parsimonious explanation of the experimental evidence is provided by the classical view that the alpha rhythm reflects idling (Pfurtscheller et al., 1996) or the recent proposal that it reflects a default rhythm that prevents effective communication (Fries, 2015). Alpha rhythms within a given cortical area are reduced by appropriate sensory stimulation or by directing attention to the appropriate part of sensory input space, e.g. by attending to the contralateral visual hemi-field (Fries et al., 2008b; Haegens et al., 2012; Handel et al., 2011; Sauseng et al., 2005; van Ede et al., 2011; van Ede et al., 2014; Worden et al., 2000). Similar to the beta and the gamma rhythm, also the alpha rhythm entails rhythmic inhibition. This rhythmic inhibition is reflected in behavioral performance. Alpha phase affects the detectability of peri-threshold stimuli (Busch et al., 2009; Duguė et al., 2011; Mathewson et al., 2009), the occurrence of TMS-induced phosphenes (Romei et al., 2008) and the incorporation of predictable distracting stimuli into working memory (Bonnefond and Jensen, 2012). Pre-stimulus alpha phase modulates the evoked BOLD response to short visual stimuli (Scheeringa et al., 2011b). Alpha power also modulates the flow of task relevant and irrelevant activation from lower to higher order brain regions (Zumer et al., 2014).

**Fig. 1.**
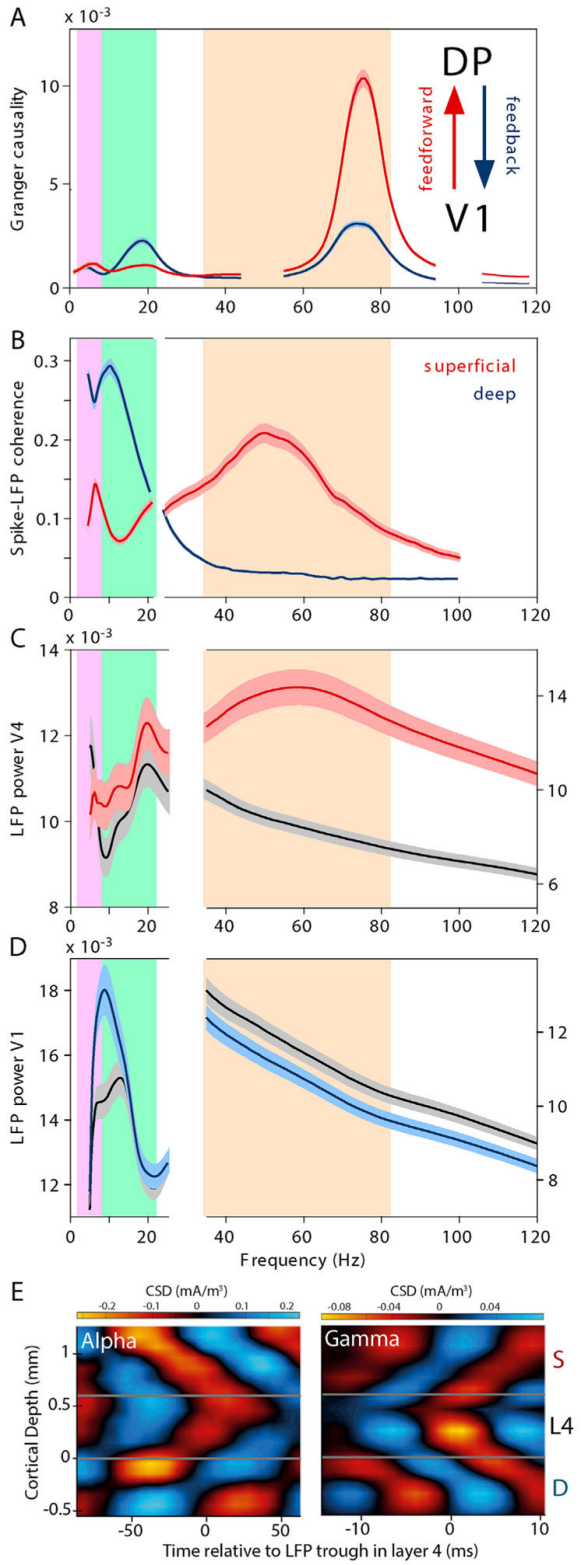
Frequency and laminar specific feedforward and feedback projections. This figure and legend was largely adapted from Fries (2015). (A) Granger-causal influences between awake macaque areas V1 and DP. The influence in the V1-to-DP direction is through an anatomical feedforward-type projection and predominates in the theta and gamma bands, indicated by purple and orange backgrounds, respectively. The influence in the DP-to-V1 direction is through an anatomical feedback-type projection and predominates in the beta band, indicated by green background. Figure adapted from Bastos et al. (2015b). (B) Spike-LFP coherence from awake macaque area V2, for recordings from deep (blue) and superficial (red) layers. Spike-LFP coherence shows an alpha-beta band peak for deep layers and both a theta and a gamma peak for superficial layers (adapted and modified from Buffalo et al., 2011). (C) Awake macaque V4 LFP power during visual stimulation with a background stimulus (black) and additional electrical stimulation in V1 (five pulses at 200 Hz), which leads to power enhancement in the gamma band (red). (D) Awake macaque V1 LFP power during visual stimulation with a background stimulus (black) and additional electrical stimulation in V4 (five pulses at 200 Hz), which leads to power enhancement in the alpha-beta band (blue). Analysis of current source density (CSD) derived from laminar recordings in awake monkey area V1. Laminar CSD was averaged relative to troughs in the alpha-filtered (left) and gamma-filtered (right) LFP from layer 4. The analysis reveals interlaminar alpha-band synchronization with systematic delays as a function of distance from layers 1 and 6 and interlaminar gamma-band synchronization with systematic delays as a function of distance from layer 4. (C–E) are adapted and modified from van Kerkoerle et al. (2014).

The notion that alpha and beta rhythms (in the following addressed as alpha-beta rhythms, while acknowledging that they are separate rhythms) play an important role in the top-down control of the flow of information between brain regions implies a laminar specificity of this process. Visual cortical areas are arranged in a hierarchy with characteristic laminar projection patterns (Barone et al., 2000; Felleman and Van Essen, 1991; Markov et al., 2014a, 2014b). Feedforward projections target layer 4 (Felleman and Van Essen, 1991); they originate predominantly in supragranular layers, and this preference is weak for projections traversing one hierarchical level and gets stronger for projections traversing more hierarchical levels, i.e. it is quantitatively related to the hierarchical distance (Markov et al., 2014b). Feedback projections avoid targeting layer 4 (Felleman and Van Essen, 1991); they originate pre-dominantly in infragranular layers, and again, this preference is weak for projections traversing one hierarchical level and gets stronger for projections traversing more hierarchical levels and is thereby quantitatively related to hierarchical distance (Markov et al., 2014b). Consistent with this pattern, current-source density analysis of the alpha rhythm in V1 suggests that alpha-rhythmic synaptic inputs first arrive in supragranular and infragranular layers and then progress to layer 4 (van Kerkoerle et al., 2014) (Fig. 1E).

The gamma rhythm (~30–100 Hz) gained prominence at the end of the 1980s, most notably through the work of Singer and colleagues. This early work was mainly focused on the binding-by-synchrony hypothesis, which states that gamma-band synchronization among spatially distributed cortical activity is the neuronal correlate of the perceptual binding between stimulus features (Gray et al., 1989; Singer and Gray, 1995). These studies originally used moving bars or gratings to stimulate the visual cortex in anesthetized cats, yet gamma-band activity was later also observed in visual cortex of awake animals (Friedman-Hill et al., 2000; Fries et al., 1997, 2001; Kreiter and Singer, 1996; Maldonado et al., 2000) and in numerous other brain regions (Fries, 2009). Several studies have demonstrated that gamma band rhythms can be recorded non-invasively in human subjects, e.g. in visual (Hoogenboom et al., 2006; Muthukumaraswamy and Singh, 2008, 2013; Swettenham et al., 2009), somatosensory (Bauer et al., 2006; Gross et al., 2007), and motor cortex (Ball et al., 2008; Schoffelen et al., 2005, 2011) and in parietal control areas (Medendorp et al., 2007; Van Der Werf et al., 2008, 2010). Importantly, in human subjects and several other mammalian species, gamma band synchronization has been observed outside early sensory cortices (Bragin et al., 1995; Brown et al., 1998; Kim et al., 2016; Medendorp et al., 2007; Pesaran et al., 2002; Schoffelen et al., 2011). This indicates that gamma band synchronization is not a purely sensory driven phenomenon, but reflects a general aspect of cortical function (Fries, 2009). Although gamma rhythms can probably occur across the entire cortex, gamma band synchrony exhibits great spatial specificity. For example, the first studies in early visual cortex already demonstrated selectivity for visual stimulus orientations (Gray et al., 1990), and later studies in the lateral intraparietal area observed selectivity for the direction of an upcoming saccade (Pesaran et al., 2002). Furthermore gamma-band synchronization within and between visual areas is modulated by selective attention (Bosman et al., 2012; Fries et al., 2001; Gregoriou et al., 2009; Grothe et al., 2012; Womelsdorf et al., 2006).

The finding that gamma-band synchronization reflects a general cortical mechanism, that it exhibits specificity to stimulus features, and that it is modulated by selective attention, have been integrated in the Communication-through-Coherence (CTC) hypothesis (Fries, 2005, 2015). The CTC hypothesis proposes that synchronization affects communication between neuronal groups. Central to this mechanism is that the gamma cycle reflects the alternation of periods with strong inhibition, during which neurons are less receptive to synaptic inputs, with short periods with weak inhibition, during which neurons respond stronger to inputs (Ni et al., 2016). For effective communication between brain regions, these time periods need to be aligned such that synaptic inputs consistently arrive during periods, when postsynaptic neurons are not inhibited (Besserve et al., 2015; Womelsdorf et al., 2007).

The basic predictions of the CTC hypothesis are well supported by empirical evidence: neuronal spiking within an activated group of neurons is typically coupled to the gamma phase (Gray et al., 1989), gamma band rhythms in different brain areas can be coherent to each other (Gregoriou et al., 2009), and this interareal coherence is strongly modulated by selective attention in the way predicted by the CTC hypothesis (Bosman et al., 2012; Grothe et al., 2012). The latter point was supported by a study that measured ECoG from a large part of the macaque cortex, while monkeys performed a selective attention task, in which they attended to one of two visual stimuli and ignored the other (Bosman et al., 2012). While these two stimuli activated separate neuronal groups in V1, they activated partially overlapping groups of neurons in V4. These V4 neurons showed gamma-band synchronization primarily with the V1 neurons activated by the attended stimulus. When selective attention switched from one stimulus to the other, so did the interareal gamma-band coherence. Analyses of directed influences using Granger Causality revealed that gamma band synchronization between V1 and V4 was primarily feedforward directed. This observation indicated that communication through coherence might not operate in the gamma-frequency band for all directions of communication between brain regions (Fig. 1A). In the meantime, several studies have demonstrated that gamma-band coherence subserves communication primarily in the feedforward direction and less in the feedback direction (Bastos et al., 2015b; Michalareas et al., 2016; van Kerkoerle et al., 2014).

Laminar electrophysiological recordings support the notion that gamma band synchronization subserves communication in the feedforward direction. Neuronal spiking is locked to gamma primarily in the supragranual layers, which are the main source of feedforward projections (Buffalo et al., 2011) Fig. 1B). Gamma-band coherence between macaque areas V1 and V2 corresponds closely to the laminar pattern of the corresponding anatomical feedforward projections from layers 2/3 to layer 4 (Roberts et al., 2013). Also electrical microstimulation supports this notion (van Kerkoerle et al., 2014). When awake macaque V1 is electrically stimulated, this elicits gamma-band activity in V4, through feedforward influences from V1 to V4. By contrast, when the same electrical stimulation is applied to area V4, this does not elicit gamma-band activity in V1; rather, it enhances alpha-band activity, if the respective V1 region is at the same time visually stimulated with a background pattern. The same study employed laminar recordings in macaque V1 and found that gamma- and alpha-band activity propagate differently across the layers: While gamma-band activity propagates from layer 4 to both supra- and infragranular layers, alpha-band activity propagates in the opposite direction, from supra- and infragranular layers to layer 4. As mentioned before, feedforward inputs arrive primarily in layer 4, whereas feedback inputs arrive primarily in layers 1 and 6. Thus, the inter-laminar propagation patterns found for gamma and alpha are consistent with their predominant roles in feedforward and feedback processing, respectively. Some of these results by van Kerkoerle et al. (2014) are shown in Fig. 1 C-E.

These studies indicate that communication across the visual hierarchy is subserved by frequency specific interareal synchronization: Communication along feedforward projections is mediated by gamma-band synchronization and communication along feedback projections by alpha-beta-band synchronization. This notion suggests that the cortical hierarchy reflects the pattern of frequency-specific directed influences between brain regions. Bastos et al. (2015b) demonstrated that this is indeed the case by reconstructing the hierarchical relationship between eight visual brain regions in macaques from directed influences obtained with ECoG data. The functional hierarchy based on the pattern of frequency-wise Granger-causality during visual stimulation and task performance was nearly identical to the anatomical hierarchy based on retrograde tracing (Barone et al., 2000; Felleman and Van Essen, 1991; Markov et al., 2014a, 2014b). The relative strength of anatomical feed-forward feedback projections correlated with the relative strength of feedforward and feedback influences in the three frequency bands. Interestingly, this study also revealed that the functional hierarchy reconstructed from ECoG data can change dynamically, e.g. with visual stimulus onset and task engagement. This indicates that although the hierarchical relation between brain regions is affected by anatomical connections, the actual neuronal interactions reflected in frequency-wise directed influences allow for the flexibility that is necessary for changing task demands. While this original demonstration of a Granger-causality based functional hierarchy was based on monkey ECoG data, a subsequent study by Michalareas et al. (2016), replicated and extended this to source-level MEG recorded in humans.

Together, the studies discussed in this section clearly demonstrate that different cortical layers are associated with different roles in neuronal communication for alpha-beta and gamma band synchronization. In recent theories of cortical function, these notions have been included. In an updated version of the Communication-through-Coherence hypothesis (Fries, 2015), gamma-band synchronization between brain regions reflects the feedforward stimulus-driven entrainment of postsynaptic neurons in higher order brain regions, while alpha-beta-band synchronization reflects feedback processes, that modulate the feedforward entrainment (Richter et al., 2017). Within the predictive coding framework, the same superficial-layer gamma-band synchronization is thought to reflect the computation of prediction errors that are relayed to higher order brain regions, while deep-layer beta-band synchronization is thought to reflect the projection of predictions from higher to lower order regions (Bastos et al., 2012).

Note that there is also a feedforward pathway from layer 5 of the lower cortical area, via the pulvinar (a higher order thalamic nucleus) to the higher cortical area (Sherman, 2016). Pulvinar-cortical synchronization has been reported both in the alpha and gamma band (Saalmann et al., 2012; Zhou et al., 2016). It is possible that the origin of this feedforward pathway in layer 5 corresponds to a weak but distinct gamma peak in layer 5 (Xing et al., 2012), yet this requires further investigation.

## Linking hemodynamic and electrophysiological signals

The link between electrophysiological and hemodynamic signals was investigated early-on by analyzing the timing and location of the earliest effects of spatial attention during combined electroencephalography (EEG) and cerebral blood flow measurements using positron emission tomography (PET) (Heinze et al., 1994). The event-related potential computed from the EEG revealed the attentional process with milli-second procession. Subsequent dipole modelling of this effect revealed a likely source location that corresponded with the attention effect observed with PET, suggesting a direct link between hemodynamic and electrophysiological signals. Since this work, several studies used a similar approach by comparing the source estimates of either EEG (Linden et al., 1999; Opitz et al., 1999; Wibral et al., 2008) or MEG (Dale and Halgren, 2001; George et al., 1995; Liu et al., 1998; Moradi et al., 2003; Phillips et al., 2002; Woldorff et al., 1999) features with the location of fMRI activations.

A seminal study provided a direct comparison between hemodynamic and electrophysiological recordings by measuring LFP and multi-unit activity (MUA) concurrently with the fMRI-BOLD signal from the same patch of cortex in primary visual cortex of anesthetized monkeys (Logothetis et al., 2001). By varying the duration and contrast of visual stimulation, the study disentangled the relation of the BOLD signal to MUA and LFP. This revealed that the BOLD response is better predicted by the LFP than by the MUA rate. It suggests, that the BOLD signal is more closely related to the metabolic consequences of postsynaptic currents, reflected by the LFP, than to the number of output spikes. As an example, multi-unit firing rate during sustained visual stimulation often dropped back to baseline after an initial increase, while the strength of both LFP power and the BOLD response remained elevated and followed the duration of visual stimulation, reflecting ongoing processing.

**Fig. 2.**
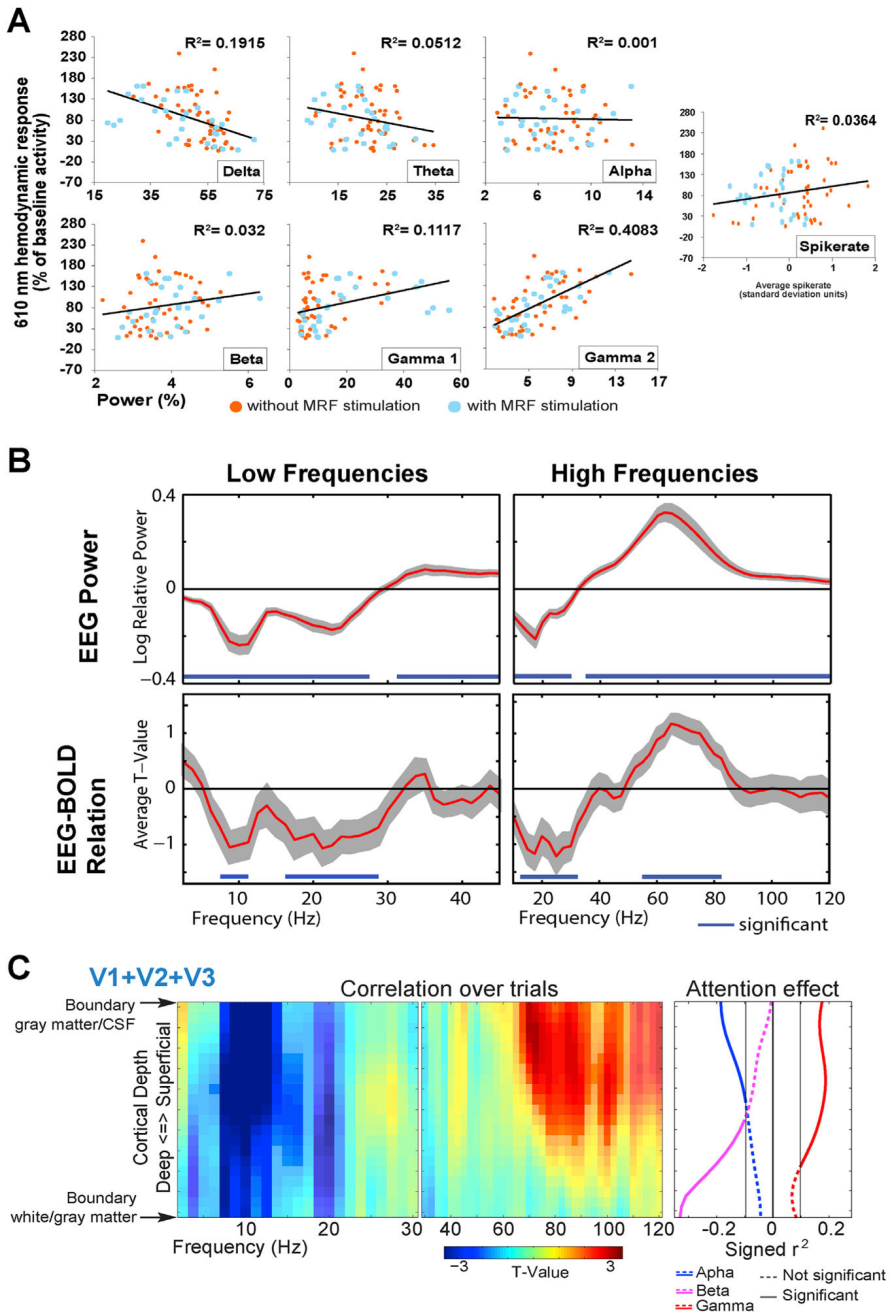
The relation between oscillatory electrophysiological activity and the BOLD signal. (A) The relationship of LFP power and spike rate with hemodynamic responses in anesthetized cat visual cortex during visual stimulation (with and without stimulation of the mesencephalic reticular formation, MRF, as indicated by dot colors). Adapted and modified from Niessing et al. (2005). Delta: 0–3 Hz; theta: 4–8 Hz; alpha: 9–14 Hz; beta: 15–21 Hz; gamma1: 22–48 Hz; gamma2: 52–90 Hz. (B) Upper row: Human EEG power changes due to visual stimulation. Power spectra are based on multi-taper analysis with ±2.5 Hz (low frequencies) and ±10 Hz (high frequencies) frequency smoothing. Lower row: Same data as upper row, but showing the trial-by-trial correlation between EEG power and BOLD signal in human visual cortex during a visual attention task. Adapted and modified from Scheeringa et al. (2011a). (C) Cortical depth-resolved relation between EEG power and the BOLD signal in human visual cortex during a visual attention task, in which subjects had to detect a stimulus change depending on whether a cue indicated whether a change was likely or would not occur. Frequency analysis as for panel B. Left: The trial-by-trial correlation between EEG power and BOLD signal. Right: The correlation between attention affects in EEG power and BOLD across subjects for alpha, beta and gamma power. Adapted and modified from Scheeringa et al. (2016).

The relation of the BOLD signal to the LFP and to MUA firing rates was further investigated in several studies (Goense and Logothetis, 2008; Niessing et al., 2005; Shmuel et al., 2006; Viswanathan and Freeman, 2007). All of these studies combined concurrently recorded electro-physiological (LFP and MUA) and hemodynamic signals (through optical imaging or MRI) in primary visual cortex of anesthetized or awake cats or macaques, and all replicated that LFP better predicts the BOLD signal than MUA. Several of these studies quantified the LFP-BOLD relation separately for different frequency bands (Goense and Logothetis, 2008; Niessing et al., 2005; Viswanathan and Freeman, 2007). They observed that the strongest link between LFP power and the hemodynamic response existed for power in the gamma-frequency band. Fig. 2A shows the respective results from one of these studies (Niessing et al., 2005). Interestingly, for the delta and theta frequency bands, these authors find a negative relation between LFP power and the BOLD signal: higher LFP power was associated with a lower hemodynamic response.

The negative relation between BOLD and low frequency field potential power is also reflected in recordings from the human brain. Several studies involving concurrently recorded EEG and fMRI (Laufs et al., 2003; Moosmann et al., 2003; Scheeringa et al., 2008, 2011a, 2009; Yuan et al., 2010; Zumer et al., 2010), but also some relating separately recorded MEG and fMRI (Zumer et al., 2010), have demonstrated a negative correlation between the cortical BOLD signal and the strength of theta, alpha, and beta rhythms in both resting state as well as task contexts. Thus, studies in human subjects related the BOLD signal primarily to decreases in electrophysiological low-frequency components, whereas studies in anesthetized animals related the BOLD signal primarily to increases in electrophysiological gamma-frequency components.

To investigate whether electrophysiological gamma-band activity shows a positive correlation to the BOLD signal in awake human subjects, we conducted an experiment with concurrently measured EEG and fMRI (Scheeringa et al., 2011a). We used a visual attention paradigm, during which subjects had to detect an increase in speed in an inward contracting circular grating. This is a well-studied paradigm that reliably induces decreases in alpha- and beta-band power and pronounced increases in gamma-band power. Previous MEG recordings demonstrated that gamma-band activity during this task originates in early visual cortex (Hoogenboom et al., 2006, 2010). Furthermore, the gamma-band increase in human visual cortex was very similar to increases observed in early visual cortex of awake monkeys during similar tasks (Bosman et al., 2012; Buffalo et al., 2011; Fries et al., 2008a). We observed, that variability over trials in the alpha, beta and gamma responses correlated with variability in the BOLD signal (Fig. 2B). In line with previous work in human subjects, alpha- and beta-band variability correlated negatively with the BOLD signal, and in line with previous invasive recordings in animals, gamma-band activity correlated positively with the BOLD signal. Importantly, across-trial variability in gamma power was not correlated to across-trial variabilities in alpha or beta power. This indicates that the neuronal processes underlying on the one hand alpha- and beta- and on the other hand gamma-band synchronization contribute to the BOLD signal in independent ways.

The finding that alpha-beta and gamma band synchronization reflect distinct neuronal processes, which contribute independently to the BOLD response, corresponds well with their different roles in information processing described in the previous section of this review. In this section, we also discussed how these rhythms are linked to layer-specific interareal anatomical projections. These notions were integrated in an experiment that built on the study described in the last paragraph (Scheeringa et al., 2011a) and that used concurrently recorded EEG and layer-specific fMRI (Scheeringa et al., 2016). This experiment aimed at describing the cortical depth-resolved profile of the relation between EEG power and the BOLD signal for different frequency bands. The experimental paradigm was adapted from the previous experiment, with as main difference the inclusion of a cue that predicted whether a visual speed increase was likely or would not occur at all. This crude attention modulation made it possible to not only correlate EEG power and BOLD fluctuations across trials, but also to correlate respective attention effects across subjects. The analysis revealed layer-specific correlational profiles for the alpha, beta and gamma bands (Fig. 2C). In line with laminar recordings revealing current sources for the alpha band in both deep and superficial layers (Bollimunta et al., 2008, 2011; Haegens et al., 2015; van Kerkoerle et al., 2014), alpha power variability across trials was negatively correlated to the BOLD signal at all cortical depths. The correlation of the attention effects however was limited to superficial layers, suggesting separate deep and superficial layer alpha-band related processes that can respond differentially to task manipulations. For the beta band, a negative correlation of attention effects across subjects was observed in the deep layers. This location corresponds to the putative role that beta plays in feedback processing mediated through deep-layer projections (Bastos et al., 2012; Fries, 2015). For the gamma band, both, variability in power over trials and variability in the attention effect over subjects, were positively correlated to BOLD variability in middle and superficial layers.

In summary, the works reviewed in this section provide ample evidence that changes in the BOLD signal differentially relate to power changes in electrophysiological measures in different frequency bands. As discussed in the previous section, these different frequencies have been linked to different roles in neuronal communication between regions, which in turn are related to different laminar projections. Largely in line with these roles, alpha, beta and gamma band rhythms were found to relate to the BOLD signal at different cortical depths. Together, these observations indicate that laminar fMRI can potentially complement investigations in the roles these rhythms play in information processing.

## Applications for laminar fMRI

**Fig. 3.**
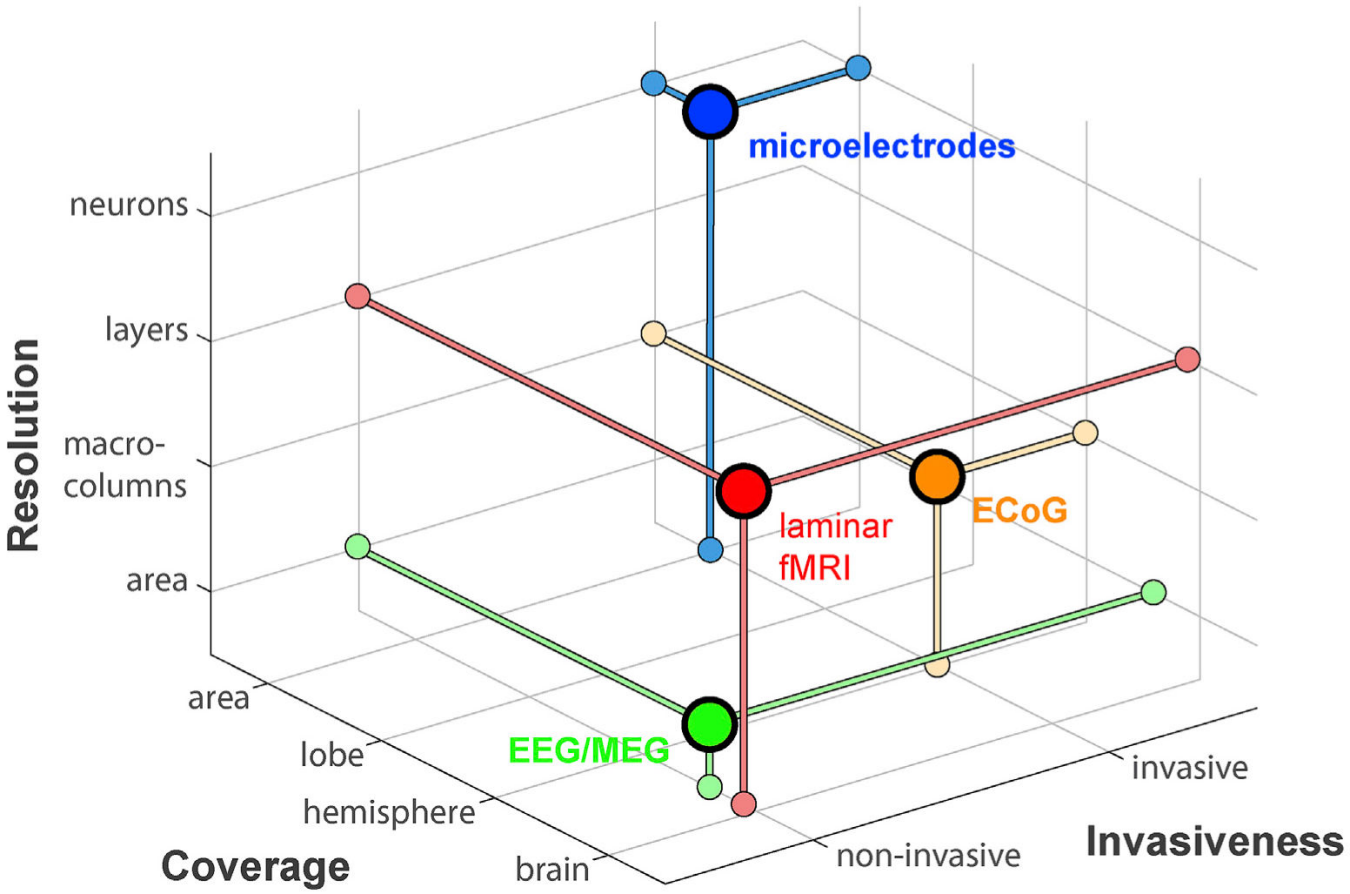
Schematic ordering of functional measurement techniques in terms of resolution, coverage and invasiveness. EEG/MEG are non-invasive, cover close to the entire brain but are limited in terms of resolution, which is restricted to the level of brain regions. The resolution of ECoG is at the level of macro-columns and can potentially cover up to roughly one hemisphere, but is a highly invasive technique. Like ECoG, micro-electrodes are also invasive, and typically limited to one or a few areas in coverage, but uniquely allow direct measurements of laminar electrophysiological activity. Although fMRI is an indirect measure of neural activity, its resolution allows measurement of laminar activity non-invasively, and it can in principle cover the whole brain, if operated at the limits of current technology.

The research with electrophysiological methods presented in this review is primarily focused on the role of rhythms in shaping the flow of information through cortex. A variety of electrophysiological recording techniques, ranging from single micro-electrodes and laminar electrodes measuring LFP and neuronal spiking to large scale ECoG and MEG recordings have been used to study this process. For laminar fMRI to be a valuable tool in this research area, it should provide us with information that cannot easily be obtained by any of these other techniques. For this purpose, it is insightful to organize the properties of electrophysiological recordings and laminar fMRI on three axes (Fig. 3): The degree of invasiveness (non-invasive and invasive), the spatial coverage (area, lobe, hemisphere, brain) and the spatial resolution (neurons, layers, areas).

Of the techniques mentioned above, the most direct way to electro-physiologically record neuronal activity in a layer-specific way is the insertion of laminar probes into cortex, i.e. probes with multiple recording contacts that measure LFP and neuronal spiking at multiple sites across the depth of cortex. By measuring with laminar probes simultaneously in more than one brain area, a detailed frequency and layer specific understanding of directed interareal influences can be obtained (Roberts et al., 2013). This method thus allows for the most fine-grained and direct assessment of the laminar pattern of interareal synchronization. The number of areas that can be measured simultaneously however is currently technically limited. Therefore, it is currently not feasible to characterize interactions in a network of multiple brain areas spanning a large part of the cortex. For such studies, ECoG is better suited to characterize network-wide frequency-specific neuronal interactions (Bastos et al., 2015b; Lewis et al., 2015; Rubehn et al., 2009). By using subdural ECoG recordings, a precisely localized neuronal response can be recorded (Bosman et al., 2012; Rols et al., 2001). ECoG however does not allow direct assessment of the laminar origin of the measured signal. Another disadvantage is that ECoG and laminar recordings are invasive recording techniques, and therefore limited to intracranial recordings in animals and occasional measurements in patients with electrodes implanted for clinical reasons (Cash et al., 2009; Csercsa et al., 2010; Ulbert et al., 2004). For direct neurophysiological recordings in healthy human subjects, EEG and MEG are currently the most widely used options. With both EEG and MEG, signals across a major part of the cortex can be measured, and especially with source-reconstructed MEG, frequency specific interactions across a wide variety of brain regions can be investigated (Michalareas et al., 2016). Although recent studies explore the possibility to localize neuronal sources of MEG signals with laminar resolution (Troebinger et al., 2014), the spatial accuracy of EEG and MEG in regular recordings is not sufficient for this and is typically substantially below the resolution of ECoG. In contrast, laminar fMRI allows for non-invasive recordings in healthy subjects at sub-millimeter resolution from multiple brain regions simultaneously. While laminar fMRI does not reach the layer-wise resolution of electrophysiological laminar probes (with typical inter-contact spacing of 50–200 μm), it does provide the most crucial distinction between supragranular and infragranular compartments. In terms of spatial extent and accuracy, laminar fMRI overlaps with the coverage provided by both laminar electrodes, ECoG and MEG/EEG. At the limits of current technology, highly accelerated EPI sequences at high field strengths (7T or larger) allow in principle for the recording of whole brain activity with a resolution of 1 mm or better in approximately 4 s (Polimeni et al., 2010; Zahneisen et al., 2015). Laminar fMRI therefore combines the ability of assessing layer-specific activity, otherwise only possible with invasive laminar recordings, with the ability to record from many cortical areas simultaneously.

The applicability of laminar fMRI necessitates taking into account layer-specific hemodynamic effects and possible confounds (Goense et al., 2016). For example, the venous blood flows from deep to superficial layers, resulting in deep-layer effects to spread into superficial layers. The extent, to which this effect is present, is in part dependent on the fMRI sequence used. For example, with spin-echo sequences, the relative venous contribution to the BOLD signal is less, compared to gradient echo sequences (Parkes et al., 2005; Yacoub et al., 2003), yet this generally comes at the cost of increased echo times and a longer acquisition per volume. These effects are extensively described and modeled in Uludağ et al. (2009). Another strategy is to formulate a cortical-depth-dependent hemodynamic model, that adequately accounts for and removes the venous drainage effects (Heinzle et al., 2016; Markuerkiaga et al., 2016). An additional challenge is posed by differences in capillary density across cortical depth (Lauwers et al., 2008). These potentially confounding factors can be particularly problematic when studying interareal laminar connectivity, as discussed in this article in relation to frequency-specific interareal synchronization, and they need to be addressed with diligence.

Analysis of regular fMRI can be divided into roughly three methodological approaches: (1) activation studies, that investigate which brain regions relate to specific experimental contrasts, (2) network-oriented approaches, that attempt to characterize the connectivity between brain regions in either task or resting state contexts, and (3) (multivariate) decoding approaches, that attempt to decode the stimuli/task conditions from the spatial pattern of the measured fMRI responses. Each of these approaches can also be applied at the laminar level, even though the measurement of laminar fMRI can pose additional challenges in the analysis (Markuerkiaga et al., 2016; Waehnert et al., 2014). FMRI studies that employ either an activation approach (De Martino et al., 2015; Kok et al., 2016) or a decoding approach (Muckli et al., 2015) at the laminar level to study cortical functioning have now been published, while network-oriented studies are still under way at the moment. In principle, all three approaches can complement investigations of brain rhythms, and we will further illustrate this below by suggesting potentially fruitful directions for each of these approaches.

Gamma and alpha-beta band activity has been linked to interareal feed-forward and feed-back influences, respectively, which are in turn linked to separate layer-specific anatomical projections. Successful independent manipulation of either feed-forward or feed-back influences should result in layer-specific activation patterns, which can be tested by laminar fMRI. This approach has been used in a study that modulated top-down influences by presenting subjects with Kanizsa triangles. Kanizsa triangles are composed of several Pac-Man-like shapes (solid circles with 60 deg wedges cut out, like 83% pie charts), which form an illusory triangle when arranged appropriately (Kok et al., 2016). The presence of such an illusory triangle can be decoded from V1, suggesting top-down projections from higher order visual regions to V1. These projections were hypothesized to end in infragranular layers of V1, resulting in a stronger BOLD response compared to conditions that did not elicit an illusory triangle. The observed activations matched these predictions. Based on the electrophysiological studies presented above, this deep-layer activity can be expected to be related to deep-layer alpha-beta band activity. Since alpha-beta band power negatively correlates with the BOLD signal (Scheeringa et al., 2011a), these results could therefore directly relate to the role these frequencies have in top-down modulation of early sensory cortices.

Over the course of the past decade, multivariate decoding techniques on fMRI data gained substantially in popularity (Haxby, 2012; Haynes and Rees, 2006) and have recently also been applied to laminar fMRI data. One study used an occluder to remove feedforward input to a subregion of V1 and to investigate the contextual feedback-related activity (Muckli et al., 2015). Multivoxel pattern analysis techniques revealed that contextual information was maximal in supragranular layers, compared to a mid-layer maximum for the condition in which the mask was absent. These results suggest that contextual feedback primarily targets superficial layers. How the superficial-layer contextual feedback relates to specific frequencies could potentially be studied by investigating how laminar fMRI decoding relates to frequency-specific interareal influences assessed with MEG or simultaneously recorded EEG. The study by van Kerkoerle et al (van Kerkoerle et al., 2014). suggests that alpha-band feed-back signals target both, superficial and deep layers, which is in line with alpha-BOLD correlations in both superficial and deep layers observed by Scheeringa et al. (2016).

FMRI research studying brain networks can be roughly further divided into studies investigating BOLD signal correlations, also called “functional connectivity”, and studies investigating stimulus- or task-dependent changes in functional connectivity, also called “effective connectivity” (Friston, 1994). The investigation of fMRI functional connectivity in the absence of specific stimulation or tasks have revealed several so-called resting state networks. Each of those resting state networks is characterized by relatively strong functional connectivity among its constituent nodes, compared to the functional connectivity with other brain regions. Interestingly, the topographies of these resting state networks are good predictors of co-activation patterns observed during task performance (Smith et al., 2009). Furthermore, different resting state networks have been linked to specific EEG/MEG frequency bands (Hipp et al., 2012; Mantini et al., 2007; Scheeringa et al., 2008, 2012). Source reconstructed MEG resting state measurements have demonstrated that networks with a similar topography as observed in resting state fMRI can best be constructed from spontaneous power fluctuations in the alpha-beta band (Brookes et al., 2011). As interareal alpha- and beta-band Granger causality are linked to layer-specific anatomical feedback projections, (cortical) resting state network connectivity might reflect primarily feedback projections between anatomically connected brain regions. ECoG recordings in monkeys however indicated that high frequency gamma-band connectivity also plays a role in resting state connectivity (Lewis et al., 2016). Future resting state fMRI studies with laminar resolution might therefore help us understand how resting state networks form. Resting state networks, whose BOLD signal correlations are mainly driven by infragranular layers would suggest a predominant role of feedback projections, whereas BOLD signal correlations mainly driven by supragranular layers would suggest an important role of feedforward projections (if the respective anatomical projection rule applies, like e.g. among visual areas). It is conceivable that supra-and infra-granular layers of a given area show different spatial patterns of BOLD signal correlation and thereby participate in different resting state networks.

An important approach to study connectivity in a task context is dynamic causal modelling (DCM). DCM allows comparing formal models of connected brain regions hypothesized to be relevant for the task. Among several such models, this approach selects the most likely model given the data. DCM has been developed for both fMRI (Friston et al., 2003) as well as electrophysiology (Bastos et al., 2015a; Friston et al., 2012; Pinotsis et al., 2014). The DCM approach recently introduced layer-specific neuronal populations, both for electrophysiological data (Bastos et al., 2015a) and also for the integration of electrophysiological data with fMRI data (Friston et al., 2017). These DCMs include different layer-specific populations and connections based on a canonical micro-circuit (Bastos et al., 2012). As a result, they allow to relate both hemodynamic and electrophysiological measures (both ERPs and frequency specific induced responses) to these different neuronal populations. In its current form, hemodynamics have not been specified at the laminar level. If this were added in future DCMs, this would allow to constrain the model both, by electrophysiological data with spectral and sometimes laminar resolution, and by fMRI data with laminar resolution.

As discussed above, high-resolution ECoG in macaques combined with Granger-causality analyses revealed a hierarchy of visual areas based on directed functional interactions, which was highly correlated to the known hierarchy based on anatomical projection patterns (Bastos et al., 2015b). A very similar functional hierarchy was found in human subjects based on source-level Granger-causality analysis of MEG data (Michalareas et al., 2016). These results suggest that feedforward projections use gamma to exert their influence, whereas feedback influences use alpha-beta. While those anatomical projections are fixed on behavioral time scales, Granger-causal influences can change dynamically. If future connectivity analyses started to use laminar fMRI, this would allow to study the laminar aspects within these hierarchically ordered networks of brain regions. A crucial question in this respect is to what extent laminar fMRI connectivity reflects the directed connectivity measures used in electrophysiology, and whether similar cortical hierarchies can be reconstructed from laminar fMRI connectivity measures. If similar hierarchies can be reconstructed, this would open up the possibility to study the functioning of these hierarchical networks at the laminar level with fMRI.

## Summary

In this review, we have given an overview of recent developments and theories about the role that neuronal rhythms play in shaping brain function, and how these rhythms are linked to laminar anatomical projection patterns. Since the strength of neuronal rhythms is coupled to the laminar BOLD signal, laminar fMRI has the potential to play an important role in investigating the role neuronal rhythms play in brain function. Although hemodynamic measures provide a metric of neural activity that is slow and indirect, laminar fMRI uniquely provides a non-invasive layer-specific metric with potentially whole brain coverage. By suggesting possible research directions, we hope to alert researchers interested in neuronal rhythms to the opportunities that laminar fMRI offers for their research.
